# Risk factors associated with severe disease in respiratory syncytial virus infected children under 5 years of age

**DOI:** 10.3389/fped.2022.1004739

**Published:** 2022-08-30

**Authors:** Lise Beier Havdal, Håkon Bøås, Terese Bekkevold, Anne-Marte Bakken Kran, Astrid Elisabeth Rojahn, Ketil Størdal, Sara Debes, Henrik Døllner, Svein Arne Nordbø, Bjørn Barstad, Elisebet Haarr, Liliana Vázquez Fernández, Britt Nakstad, Christopher Inchley, Elmira Flem

**Affiliations:** ^1^Department of Paediatric and Adolescent Medicine, Akershus University Hospital, Lørenskog, Norway; ^2^Norwegian Institute of Public Health, Oslo, Norway; ^3^Department of Microbiology, Oslo University Hospital, Oslo, Norway; ^4^Division of Paediatric and Adolescent Medicine, Oslo University Hospital Ullevål, Oslo, Norway; ^5^Department of Paediatrics, Østfold Hospital Kalnes, Grålum, Norway; ^6^Division of Paediatric and Adolescent Medicine, Institute of Clinical Medicine, University of Oslo, Oslo, Norway; ^7^Department of Medical Microbiology, Østfold Hospital Kalnes, Grålum, Norway; ^8^Department of Paediatrics, St. Olavs University Hospital, Trondheim, Norway; ^9^Department of Clinical and Molecular Medicine, Norwegian University of Science and Technology, Trondheim, Norway; ^10^Department of Medical Microbiology, St. Olavs University Hospital, Trondheim, Norway; ^11^Department of Paediatric and Adolescent Medicine, Stavanger University Hospital, Stavanger, Norway; ^12^Department of Medical Microbiology, Stavanger University Hospital, Stavanger, Norway

**Keywords:** respiratory syncytial virus (RSV), risk factor (RF), disease severity analysis, pediatric infection, respiratory infection

## Abstract

**Objective:**

To evaluate risk factors for severe disease in children under 59 months of age hospitalized with respiratory syncytial virus (RSV) infection.

**Study design:**

We prospectively enrolled 1,096 cases of laboratory confirmed RSV infection during three consecutive RSV seasons in 2015–2018. Potential risk factors for severe disease were retrieved through patient questionnaires and linkage to national health registries. Need for respiratory support (invasive ventilation, bi-level positive airway pressure, or continuous positive airway pressure), and length of stay exceeding 72 h were used as measures of disease severity. Associations were investigated using multivariable logistic regression analyses. Multiple imputation was used to avoid bias and inference induced by missing data.

**Results:**

Risk factors associated with a need for respiratory support included age younger than 3 months of age [aOR: 6.73 (95% CI 2.71–16.7)], having siblings [aOR: 1.65 (95% CI 1.05–2.59)] and comorbidity [aOR: 2.40 (95% CI 1.35–4.24)]. The length of hospital stay >72 h was significantly associated with being younger than 3 months of age [aOR: 3.52 (95% CI 1.65–7.54)], having siblings [aOR: 1.45 (95% CI 1.01–2.08)], and comorbidity [aOR: 2.18 (95% CI 1.31–3.61)]. Sub-group analysis of children younger than 6 months of age confirmed the association between both young age and having siblings and the need for respiratory support.

**Conclusion:**

In a large cohort of children <59 months hospitalized with RSV infection, young age, comorbidity, and having siblings were associated with more severe disease.

## Introduction

Respiratory syncytial virus (RSV) is the most common pathogen identified in pediatric patients presenting with lower respiratory tract infection, which imposes a substantial medical burden among children under 5 years of age worldwide (1–3). The estimated RSV attributable mortality in this age-group is as high as 118,200 annual deaths (1), with a clear majority of deaths occurring in developing countries. By the age of two, almost all children have been infected with RSV (4, 5). While most children experience a rather mild disease course, some are prone to more severe illness. Preterm infants and children with chronic medical conditions such as chronic lung disease, hemodynamically significant congenital heart disease, immune deficiency, neuromuscular impairment, and trisomy 21, are susceptible to a severe course of RSV illness (6–11). Nevertheless, the majority of severe RSV cases occur among otherwise healthy full-term infants (2). Factors associated with severe cases have been the subject of epidemiological and immunological research for decades (7, 12–14). A number of environmental, host-related, and gestational factors are cited as risk factors for severe outcome of RSV infection in previously healthy children, including male sex, low socioeconomic status, siblings, day care attendance, lack of breast feeding, low birth weight, and family history of atopy (7, 8).

The high incidence of RSV infection imposes a substantial burden on the healthcare system (2). This emphasizes the importance of identifying children at high risk for severe outcome, to prioritize such populations for preventive treatment. With the current promising results in the development of vaccines and new monoclonal antibodies protecting against RSV disease, identification of the appropriate target population for prophylaxis is more important than ever (15, 16).

The aim of this study was to further explore risk factors predisposing for more severe disease in Norwegian children under 5 years of age hospitalized for RSV infection, and in the sub-group of infants younger than 6 months of age.

## Materials and methods

### Sample

The Norwegian Enhanced Pediatric Immunization Surveillance (NorEPIS) is a network consisting of five hospitals with a combined catchment population approaching 44% of the pediatric population in Norway. NorEPIS implemented active prospective RSV surveillance in children under 5 years of age from 2015 to 2018. Surveillance was conducted during the winter season, defined as the period from week 40 to week 20 the following year. A detailed description of inclusion and exclusion criteria has been provided previously (2). Overall, the NorEPIS study cohort included 2,590 children aged 0–59 months from whom a nasopharyngeal flocked swab or aspirate was collected within 72 h post-enrolment. The routine for respiratory pathogen detection at all participating hospitals were real-time polymerase chain reaction (PCR). All collected nasopharyngeal samples were analyzed using the standard procedure for PCR at each of the five laboratories. The current study included data from children who tested positive for RSV by PCR. Clinical data and information about healthcare use was collected though a standardized questionnaire.

Individual patient data collected at the hospitals were subsequently linked to national health registries using unique personal identification numbers. The linkage included the Norwegian Patient Registry (NPR), which contains information on all hospital visits in Norway, including International Classification of Diseases (ICD-10) diagnoses (17). The Norwegian Primary Care Registry contains International Classification of Primary Care (ICPC-2) or ICD-10 diagnoses from all publicly funded general practitioners and primary care emergency clinics (18). Together, NPR and Norwegian Primary Care Registry cover all governmental-funded health care in Norway. The Medical Birth Registry of Norway (MBRN) contains information on gestational age, congenital malformations and disorders for all children born in Norway (19). The Norwegian Prescription Database (20) contains data on dispensed drugs in Norway, including palivizumab. A detailed description of all information retrieved from the above registries for identifying underlying risk groups has been provided elsewhere (2).

### Outcome

To assess disease severity, two outcome measures were used. The primary outcome was need for respiratory support either in the form of invasive ventilation, bi-level positive airway pressure, or continuous positive airway pressure. The secondary outcome was length of stay (LOS) measured in hours and dichotomized as stay under or above 72 h.

### Exposures

Demographic and clinical variables to be considered in the analysis were selected based on existing literature (6, 8, 9) and expert opinion. An overview of the variables of interest is given in Table 1.

**Table 1:** Summary of the variables in the association analysis including the amount of data available for each variable.

**Variable of interest**	**Definition**	**Available data for children 0–59 months of age*, n* = 1,096**	***N* (%)^a^**
**Outcome variable**			
Need for respiratory support	Treatment with invasive or non-invasive positive pressure ventilation during RSV hospitalization	1,075 (98.1%)	Yes = 202(19%)
Hospitalization > 72 h	Length of stay measured in hours and dichotomized as stay under 72 h or above 72 h.	978 (89.2%)	>72 h = 317 (28.9%)
**Demographic risk factors**			
Age	In months, by age group	1,096 (100%)	<3 m = 301 (27%) 3–5 m = 181 (17%) 6–11 m = 170 (16%) 12–23 m = 297 (27%) 24–59 m = 147 (13%)
Sex	Biological sex, male or female	1,096 (100%)	Male = 615 (56%)
Siblings	Children <18 years of age, living in the same household	1,031 (94%)	Yes = 766 (74%)
Pets	Daily contact with pet animals	1,032 (94%)	Yes = 259 (25%)
Day-care attendance	Attending nursery-home etc.	822 (75%)	Yes = 493 (60%)
**Gestational risk factors**			
Maternal age	Maternal age at time of delivery	1,091 (99.5%)	Mean = 31.0 (SD 4.90)
Smoking during pregnancy	Smoking any time during pregnancy	974 (88.8%)	Yes = 32(3%)
Born before peak season	Being born before peak of first RSV season, applies to children <6 months of age	n.a.	
Prematurity	Gestational age <32 weeks	1,090 (99%)	Yes = 29 (3%)
Cesarean delivery	Cesarean delivery, planned or acute	1,091 (99.5%)	Yes = 222 (20%)
Multiple gestation	Twins or triplets	1,096 (100%)	Yes = 56 (5%)
Small for gestational age	Birth weight Z-score < −1.28 SD according to sex and gestational age	1,090 (99%)	Yes = 87 (8%)
**Comorbidity and dispositions**			
No breastfeeding	Never breastfeed	739 (67.4%)	Yes = 46 (6%)
Family history of atopy	History of asthma or atopic disease in first order relatives	738 (67.3%)	Yes = 125 (17%)
Comorbidity	Including trisomy 21, neuromuscular impairment, congenital heart disease, pulmonary disease, BPD, immunodeficiency, and cancer. Conditions identified by ICD-10 codes provided in supp. data	1,096 (100%)	Yes = 94 (9%)
Respiratory support during neonatal period	Treatment with invasive or non-invasive positive pressure ventilation during neonatal period. Not including treatment for RSV disease	1,096 (100%)	Yes = 84 (8%)
Viral Co-detection	Influenza A and B virus, Parainfluenza virus subtype 1-3 and Human metapneumovirus	1,019 (93%)	Yes = 76 (7%)

^a^Number of positive outcomes for each variable, and N-positive as percentage of the available data.

Information on gestational age, birth weight, placenta weight, single or plural birth and maternal smoking status during pregnancy was collected from MBRN as well as data on infant's transfer to the neonatal intensive care unit for respiratory support treatment during the neonatal period. Prematurity was defined as gestational age <32 weeks.

The questionnaire filled out by parents provided information on the family history of atopy, daily contact with pet animals, number of siblings, breastfeeding history, and day-care attendance.

Comorbidities were identified based on ICD-10 codes from previous healthcare contacts (Supplementary material 1).

All nasopharyngeal samples were analyzed by PCR for RSV, Influenza A and B viruses, Parainfluenza virus subtype 1-3, and Human metapneumovirus. Viral co-detection was defined as the presence of one of these viruses in addition to RSV.

### Statistical analysis

All statistical analyses were performed using *Stata Statistical Software: Release 16*. College Station, TX: StataCorp LLC. As a first exploration of disease severity, all exposure variables were analyzed in the univariate logistic regression for the selected outcome variables. Multivariable logistic regression analyses were conducted to evaluate associations of risk factors with each measure of severity.

To avoid bias and inference induced by missing data, we used multiple imputation (MI) to impute missing data (21–24). In order to examine whether data were missing completely at random (21, 24), the degree of missingness was explored for each covariate in the model. Further, potential associations between covariates and being a case with complete information was investigated.

Complete case analysis was conducted prior to MI. In the complete dataset, we examined whether the association between exposure and outcome varied depending on changes in age or sex by testing whether there was evidence of an interaction between age, sex and each one of the additional exposures.

The number of imputations were set to 50 in order to exceed the percentage of missing data in any one variable (25), and the coefficients were combined to obtain the final estimates for the imputed model.

To ensure congeniality between the imputation and the analysis model, all variables from the univariate analysis, including both outcome variables, were also included in the imputation model. Linear and logistic regression were used in the MI as appropriate.

As missingness in some variables was likely to condition on covariates in the analysis model (e.g., breastfeeding and age), sensitivity analysis for the imputed data was conducted for any variable with a degree of missing data exceeding 5%. Sensitivity analysis was conducted using a pattern-mixture approach, assuming a missing not at random (MNAR) mechanism of missingness for the exposure. Details of the sensitivity analysis are provided in Supplementary material 3.

## Results

A total of 1,087 children with positive RSV tests, accounting for 1,096 disease episodes, were included in the study. Of these, 202 (19%) children received respiratory support during their hospital stay. The median LOS was 33 h [IQR (4.1–94)], and 29% were admitted for >72 h.

Of all enrolled children, 399 (36%) had a complete dataset (Table 1). The outcome variable, need for respiratory support, had a low percentage of missing data (1.9%), whereas the percentage of missing data for the outcome LOS > 72 h was 10.8%. Of all 19 variables, 12 had a proportion of missing data under 1.5% (Supplementary material 2).

Children with complete datasets were more likely to be younger, did not attend daycare, had no family history of atopy, had a longer hospital stay, and were more likely to receive respiratory support during hospitalization (Supplementary material 2). This confirms that the complete case analysis could potentially be biased, supporting the use of MI.

### Univariate analysis

**Figure 1:**
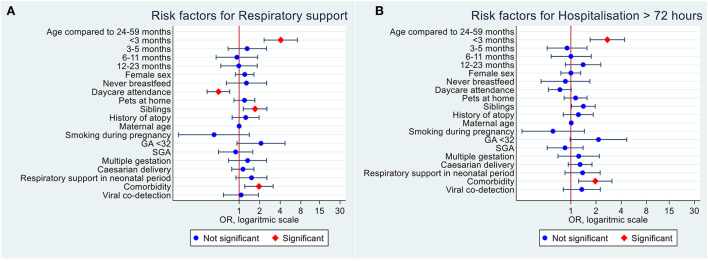
Univariate logistic regression analysis of risk factors for severe RSV infection in Norwegian children under 5 years of age. **(A)** Risk factors of need for respiratory support. **(B)** Risk factors of LOS > 72 h.

Among all RSV infected children 0–59 months of age, we found that the need for respiratory support was associated with young age, having siblings, and pre-existing comorbidities (Figure 1A). LOS >72 h was associated with young age and comorbidity status in the univariate analysis (Figure 1B).

### Multivariate analysis

In the complete case analysis among children 0–59 months of age, need for respiratory support was associated with being younger than 3 months of age [aOR: 12.5 (95% CI 2.11–73.8)], having siblings [aOR: 2.66 (95% CI 1.32–5.33)] and comorbidity [aOR: 3.19 (95% CI 1.19–8.52)]. These associations remained significant after MI (Table 2).

**Table 2:** Risk factors for need for respiratory support in RSV positive hospitalized children under 5 years of age.

	**Complete case analysis** ^**a**^		**Multiple imputation** ^**b**^	
**Risk factor**	**Adjusted OR (95% CI)**	***p*-value^c^**	**Adjusted OR (95% CI)**	***p*-value^c^**
**Demographic**				
**Age by age group**				
<3 m	12.5 (2.11–73.8)	**0.005**	6.73 (2.71–16.7)	**<0.001**
3–5 m	3.97 (0.65–24.30)	0.136	2.00 (0.77–5.17)	0.155
6–11 m	1.69 (0.27–10.76)	0.579	1.22 (0.48–3.09)	0.670
12–23 m	3.49 (0.61–19.77)	0.158	1.23 (0.64–2.37)	0.536
24–59 m	Ref		Ref	
Sex, female	0.84 (0.51–1.38)	0.485	0.79 (0.57–1.10)	0.165
Siblings	2.66 (1.32–5.33)	**0.006**	1.65 (1.05–2.59)	**0.029**
Pets at home	1.66 (0.98–2.80)	0.057	1.15 (0.78–1.68)	0.480
Day-care attendance	0.98 (0.37 to2.58)	0.964	1.21 (0.60–2.42)	0.596
**Gestational**				
Maternal age, per year	1.02 (0.97–1.07)	0.435	0.98 (0.95–1.02)	0.284
Smoking during pregnancy	0.15 (0.02–1.31)	0.086	0.42 (0.12–1.46)	0.170
GA <32 weeks	1.38 (0.21–8.91)	0.733	1.42 (0.50–4.01)	0.508
Cesarean delivery	1.78 (0.96–3.31)	0.066	1.11 (0.72–1.68)	0.643
Multiple gestation	0.83 (0.22–3.15)	0.779	1.16 (0.56–2.39)	0.696
Small for gestational age	1.01 (0.42–2.42)	0.981	0.97 (0.52–1.83)	0.935
**Comorbidity and dispositions**				
No breastfeeding	1.65 (0.65–4.17)	0.295	1.57 (0.73–3.42)	0.250
Family history of atopy	1.55 (0.75–3.19)	0.237	1.62 (0.98–2.66)	0.060
Comorbidity^d^	3.19 (1.19–8.52)	**0.021**	2.40 (1.35–4.24)	**0.003**
Respiratory disease during infancy	1.88 (0.53–6.69)	0.329	1.39 (0.69–2.78)	0.355
Viral Co-detection	1.32 (0.49–3.51)	0.581	1.32 (0.71–2.49)	0.382

^*a*^Multivariate logistic regression analysis including all co-variates listed in the table. A total of 399 cases had complete data in all covariates. ^*b*^Multiple logistic regression analysis after multiple imputation of missing data. Analysis includes all 1096 RSV cases. ^*c*^Results significant at a level of 0.05 in bold text. A total of 16 associations are explored in this table, a Bonferroni-correction for test multiplicity suggests a level of significance at 0.05/16 = 0.003. ^*d*^Comorbidity includes congenital heart disease, neuromuscular impairment, Trisomy 21, bronchopulmonary dysplasia, chronic respiratory disease excluding asthma, immunodeficiency including children receiving chemotherapy for cancer.

Sub-group analysis of the need for respiratory support in children younger than 6 months of age (Table 3) confirmed the association with young age and having siblings both in the complete case analysis and after MI. Comorbidity was only significantly associated with the need for respiratory support in the complete case analysis.

**Table 3:** Subgroup analysis of children younger than 6 months of age. Risk factors for need for respiratory support and length of stay, respectively.

	**Need for respiratory support**				**Length of stay** > **72 h**			
	**Complete Case Analysis** ^**a**^		**Multiple Imputation** ^**b**^		**Complete Case Analysis** ^**a**^		**Multiple Imputation** ^**b**^	
**Risk Factor**	**Adjusted OR (95% CI)**	**p-value^c^**	**Adjusted OR (95% CI)**	**p-value^c^**	**Adjusted OR (95% CI)**	**p-value^c^**	**Adjusted OR (95% CI)**	**p-value^c^**
**Demographic**								
**Age by age group**								
<1 m	6.86 (2.91–16.2)	**<0.001**	6.01 (3.10–11.7)	**<0.001**	7.39 (3.12–17.5)	**<0.001**	7.42 (3.90–14.1)	**<0.001**
1–2 m	3.14 (1.50–6.58)	**0.002**	3.94 (2.18–7.10)	**<0.001**	2.45 (1.21–4.94)	**0.012**	3.10 (1.80–5.35)	**<0.001**
2–3 m	1.56 (0.69–3.53)	0.287	2.05 (1.07–3.95)	**0.031**	2.07 (0.98–4.35)	0.056	2.74 (1.53–4.89)	**0.001**
3–5 m	Ref		Ref		ref		ref	
Sex, female	0.92 (0.51–1.65)	0.781	0.81 (0.52–1.27)	0.366	1.10 (0.63–1.93)	0.739	0.94 (0.61–1.43)	0.765
Siblings	2.44 (1.04–5.72)	**0.039**	2.72 (1.31–5.64)	**0.007**	1.41 (0.67–2.99)	0.367	2.25 (1.18–4.27)	**0.014**
Pets at home	1.80 (0.96–3.36)	0.065	1.56 (0.95–2.56)	0.082	1.40 (0.75–2.60)	0.287	1.59 (0.98–2.59)	0.061
Day care attendance	0.93 (0.23–3.77)	0.921	0.97 (0.30–3.11)	0.956	0.68 (0.17–2.72)	0.586	0.60 (0.20–1.82)	0.364
**Gestational**								
Maternal age, per year	1.00 (0.94–1.06)	0.987	0.97 (0.92–1.02)	0.208	1.01 (0.95–1.07)	0.804	0.98 (0.93–1.02)	0.325
Smoking during pregnancy	0.22 (0.02–2.14)	0.192	0.37 (0.07–1.93)	0.239	0.43 (0.07–2.61)	0.360	0.45 (0.11–1.91)	0.282
Born before peak season	1.26 (0.70–2.27)	0.440	1.07 (0.68–1.68)	0.769	0.96 (0.55–1.70)	0.901	1.21 (0.79–1.85)	0.385
GA <32 weeks	0.77 (0.05–11.1)	0.844	1.52 (0.17–13.4)	0.708	0.43 (0.03–6.02)	0.534	0.39 (0.04–4.05)	0.431
Cesarean delivery	2.05 (0.97–4.37)	0.062	1.48 (0.83–2.64)	0.183	1.40 (0.67–2.96)	0.374	1.31 (0.75–2.29)	0.334
Multiple gestation	1.27 (0.23–6.97)	0.786	1.37 (0.43–4.35)	0.590	0.71 (0.14–3.71)	0.684	0.67 (0.21–2.18)	0.503
Small for gestational age	1.13 (0.40–3.22)	0.820	0.92 (0.38–2.21)	0.845	1.62 (0.61–4.32)	0.337	1.20 (0.54–2.69)	0.651
**Comorbidity and dispositions**								
No breastfeeding	1.50 (0.39–5.70)	0.555	2.17 (0.73–6.46)	0.162	0.77 (0.21–2.82)	0.689	1.00 (0.32–3.10)	0.998
Family history of atopy	0.89 (0.32–2.45)	0.821	1.06 (0.46–2.43)	0.894	1.70 (0.65–4.45)	0.281	1.44 (0.61–3.39)	0.400
Comorbidity^d^, any	3.76 (1.07–13.3)	**0.039**	1.98 (0.76–5.15)	0.164	3.18 (0.90–11.3)	0.073	1.88 (0.72–4.91)	0.198
Viral Co-detection	1.51 (0.42–5.47)	0.528	1.46 (0.52–4.09)	0.467	1.85 (0.54–6.39)	0.331	1.37 (0.50–3.69)	0.540
Respiratory support during infancy	1.51 (0.28–8.20)	0.630	1.40 (0.41–4.81)	0.588	2.25 (0.44–11.53)	0.332	3.78 (1.05–13.56)	**0.041**

^*a*^Multivariate logistic regression analysis including all co-variates listed in the table. A total of 399 cases had complete data in all covariates. ^*b*^Multiple logistic regression analysis after multiple imputation of missing data. Analysis includes all 1096 RSV cases. ^*c*^Results significant at a level of 0.05 in bold text. A total of 16 associations are explored in this table, a Bonferroni-correction for test multiplicity suggests a level of significance at 0.05/16 = 0.003. ^*d*^Comorbidity includes congenital heart disease, neuromuscular impairment, trisomy 21, bronchopulmonary dysplasia, chronic respiratory disease excluding asthma, immunodeficiency including children receiving chemotherapy for cancer.

In the complete case analysis, LOS > 72 h was associated with being younger than 3 months of age [aOR: 5.92 (95% CI 1.27 to 27.7)], and family history of atopy [aOR: 2.15 (95% CI 1.12–4.11)]. After MI, LOS > 72 h was significantly associated with being younger than 3 months of age [aOR: 3.52 (95% CI 1.65–7.54)], comorbidity [aOR: 2.18 (95% CI 1.31–3.61)] and having siblings [aOR: 1.45 (95% CI 1.01–2.08)]. Results for all risk factors included in the multivariate logistic regression analysis are presented in Table 4.

**Table 4:** Risk factors for length of stay over 72 h in RSV positive children under 5 years of age.

	**Complete case analysis** ^**a**^		**Multiple imputation** ^**b**^	
**Risk factor**	**Adjusted OR (95% CI)**	***p*-value^c^**	**Adjusted OR (95% CI)**	***p*-value^c^**
**Demographic**				
**Age by age group**				
<3 m	5.92 (1.27–27.7)	**0.024**	3.52 (1.65–7.54)	**0.001**
3–5 m	2.02 (0.41–9.90)	0.386	1.08 (0.49–2.40)	0.841
6–11 m	1.20 (0.25–5.89)	0.819	1.11 (0.53–2.32)	0.789
12–23 m	3.09 (0.74–12.93)	0.122	1.55 (0.94–2.56)	0.085
24–59 m	Ref			
Sex, female	0.91 (0.58–1.44)	0.699	0.96 (0.72–1.27)	0.773
Siblings	1.53 (0.87–2.69)	0.141	1.45 (1.01–2.08)	**0.043**
Pets at home	1.49 (0.92–2.40)	0.107	1.10 (0.79–1.52)	0.582
Day-care attendance	1.00 (0.42 to2.38)	0.996	1.08 (0.60 to1.95)	0.795
**Gestational**				
Maternal age, per year	1.02 (0.97–1.06)	0.516	1.00 (0.97–1.03)	0.783
Smoking during pregnancy	0.34 (0.08–1.33)	0.120	0.56 (0.23–1.39)	0.213
GA <32 weeks	0.66 (0.11–3.88)	0.650	1.43 (0.55–3.74)	0.465
Cesarean delivery	1.18 (0.66–2.12)	0.569	1.36 (0.95–1.94)	0.096
Multiple gestation	0.82 (0.24–2.80)	0.752	1.08 (0.58–2.02)	0.813
Small for gestational age	1.43 (0.66–3.10)	0.371	1.01 (0.60–1.70)	0.967
**Comorbidity and dispositions**				
No breastfeeding	1.01 (0.42–2.43)	0.987	1.07 (0.54–2.14)	0.841
Family history of atopy	2.15 (1.12–4.11)	**0.021**	1.34 (0.87–2.07)	0.188
Comorbidity^d^	2.21 (0.89–5.47)	0.086	2.18 (1.31–3.61)	**0.003**
Respiratory disease during infancy	1.61 (0.51–5.10)	0.417	1.00 (0.54–1.85)	0.994
Viral Co-detection	1.71 (0.69–4.22)	0.245	1.48 (0.86–2.53)	0.153

^a^Multivariate logistic regression analysis including all co-variates listed in the table. A total of 399 cases had complete data in all covariates. ^b^Multiple logistic regression analysis after multiple imputation of missing data. Analysis includes all 1096 RSV cases. ^c^Results significant at a level of 0.05 in bold text. A total of 16 associations are explored in this table, a Bonferroni-correction for test multiplicity suggests a level of significance at 0.05/16 = 0.003. ^d^Comorbidity includes congenital heart disease, neuromuscular impairment, trisomy 21, bronchopulmonary dysplasia, chronic respiratory disease excluding asthma, immunodeficiency including children receiving chemotherapy for cancer.

Sub-group analysis of LOS > 72 h in children younger than 6 months of age (Table 3) confirmed the findings of young age as a risk factor in the complete case analysis. In addition, after MI, respiratory support during infancy, and having siblings were associated with a longer hospital stay in children under 6 months of age.

Co-detection of other viral agents was not associated with increased disease severity, neither measured as need for respiratory support, nor as hospitalization >72 h.

Sensitivity analysis for the primary outcome, need for respiratory support, and each of the relevant exposures are presented in Supplementary material 3.

Daycare attendance was not significantly associated with the outcome in any of the main models investigated. When we applied an extreme sensitivity parameter of 10 in the MI model, daycare attendance seemed to have protective association with RSV severity. Adding sensitivity parameters to the imputation model to imitate an MNAR missingness mechanism for breastfeeding, daily contact with pet animals, and maternal smoking during pregnancy, resulted in a significant association between family history of atopy, and need for respiratory support.

## Discussion

Using a prospective cohort of children hospitalized with RSV infection, we investigated risk factors for severity measured by need for respiratory support or hospital stay exceeding 72 h. The analyses included both children younger than 5 years of age and the sub-group of children younger than 6 months. It is well established that young age is consistently associated with severe RSV disease (7–9), which is supported by the current study in both age groups.

Having siblings has been previously reported to be an important predictor for disease severity (8, 26–29), especially in younger children (28). In the current study, having siblings was strongly associated with both outcomes of severity in both children under 6 months and under 59 months of age, after MI, and with need for respiratory support in the complete case analysis of both age groups. A plausible biological explanation for this association could be that a higher viral load at exposure causes a more severe disease course. Older siblings attending daycare are likely to be a source of RSV transmission in the household (5), which may be characterized by a higher viral load at exposure vs. community transmission due to the type of contacts and duration of contacts between household members. Alternatively, parents with several children may tend to seek healthcare for younger children at a later stage, as they feel confident handling the infection at home without need for medical attention. Hospitalization practices may also play a role, but information about hospital referral patterns among children in Norway is limited (30). It is general practitioners and primary health-care emergency ward physicians who refer children for hospital admission in Norway. Such decisions are often subjective (31), and likely to be influenced by the degree of concern by parents and other non-clinical reasons (32). Day-are attendance has previously been reported as a predictor of more severe RSV infection (28). Societal structures are likely to influence the transmission patterns of infections. In Norway, due to favorable parental benefit regulations, only 4, 6% of children younger than 1 year of age are attending day-care (33). This promotes the chances that the youngest children are infected by siblings, rather than at day-care. Acknowledging the impact of local societal structures is important when considering demographic risk groups for preventive maters against RSV.

Interestingly, prematurity did not increase the risk of severe disease in our study, which is in contrast with previous findings (26, 34–36), which suggest that premature children are at risk of more severe disease. We believe that the use of palivizumab for RSV protection in our setting is likely to influence these results. Palivizumab prophylaxis in Norway is offered free of charge to all children who fulfill the following criteria (1) gestational age <32 weeks, and bronchopulmonary dysplasia defined during the first year of life as need for supplementary oxygen at 36 weeks post-menstrual age, and during the second year of life as persistent need for supplemental oxygen at home. (2) Children younger than 1 year of age with congenital heart disease of significant hemodynamic compromise or pulmonary hypertension, or significant airway abnormalities, neuromuscular impairment, or immunosuppression. (3) Children younger than 2 years of age with significant immunosuppression, or significant cardiac disease combined with pulmonary or neuromuscular disease (37). Norwegian children who fulfill the national criteria for the use of palivizumab are likely to receive the drug, and their need for medical care due to RSV infection will be accordingly reduced. Two of the premature children in our cohort did receive palivizumab during the season of hospitalization for RSV infection. The design of the current study does not provide information on children who avoided hospitalization due to the use of palivizumab, but we observed that among non-premature children hospitalized for RSV infection, ~60% were younger than 12 months of age, whereas among premature children, the same proportion was only 28% (data not shown).

Previous studies found family history of atopy to increase the risk of severe RSV disease (6, 28). In the current study, the association between atopic disease and LOS exceeding 72 h was only significant in the complete case analysis and not after MI. In the sensitivity analysis there was a significant association between need for respiratory support and atopic disposition when any sensitivity parameter was added to breastfeeding, daily contact with pet animals, and smoking during pregnancy. These findings encourage further exploration of the association between family history of atopy and disease severity in RSV infected children.

Young age, having siblings and comorbidity were significantly associated with both outcome measures of disease severity in children 0–59 months of age. These associations remained significant in all sensitivity analyses performed, even when the extreme sensitivity parameter of 10 was added.

### Strengths and limitations

A strength of the current study is the prospective data collection, and hence an RSV case definition based on viral detection by PCR. The study is further strengthened by the use of individual patient data from comprehensive population-based registries. All healthcare for children is publicly funded and free of charge in Norway, and accordingly, any comorbidity revealed prior to the RSV episode is likely to be registered in NPR. All births in Norway are also registered in MBRN.

Our findings on co-morbidities are limited by the availability of palivizumab for defined risk groups in the population studied (37). Several comorbidities have been found to be of great importance for the risk of severe disease outcome in previous studies (7–9, 38, 39). In the current study, several comorbidities were grouped together as numbers of each comorbidity were too small to observe association between disease severity and more differentiated groups of children with pre-existing medical conditions. Since monoclonal anti-RSV antibodies are recommended and free of charge for children at high risk for severe RSV infection in Norway, our findings are likely to underestimate the risk for children with pre-existing medical conditions.

Further limitations of the current study include the amount of missing data, especially on demographic variables from the study questionnaires.

A higher degree of data completeness would increase the validity of our findings. To reduce bias and inference induced by missing data, we used MI. MI models rely on the assumption that the missingness in the data is fully dependent on the observed data, called missing completely at random (40). Sensitivity analysis was applied to the MI model by imposing specific MNAR missingness mechanisms on the data in a mixed-pattern approach. For the imputed variables family history of atopy, and daycare attendance, the MNAR imitation in the imputed data resulted in change in conclusions. To interpret these findings, one must question whether the sensitivity parameter applied in the sensitivity analysis, represents a plausible departure from the MNAR mechanism.

The presence of fever as an inclusion criterion in our study was applied to children older than 12 months of age. As fever is common in RSV disease, but not pathognomonic (6, 41, 42), this could have led to a somewhat skewed inclusion of patients. However, previous studies do support the presence of fever in most children older than 12 months of age hospitalized for RSV disease (43).

To focus on the contribution of RSV infection to the clinical state of children, the analysis was adjusted for co-detection of other viral agents. As the respiratory panel of viral agents tested for differed between hospital laboratories, only the viruses included by all hospitals were included in the analyses. Detection of multiple viruses is known to be common in lower respiratory tract infection (44). Previous studies found that viral co-detection prolonged the length of hospital stay (45, 46), and one study found that co-detection of human metapneumovirus increased the risk for intensive care unit admission (47). Several other studies did not find disease severity to be associated with detection of multiple viral agents (48–50). This is in line with our findings, and in line with the conclusion in a recent review and meta-analysis (51).

### Conclusion

In this cohort of children younger than 5 years hospitalized with confirmed RSV infection in Norway, young age, comorbidity, and having siblings were associated with more severe disease.

## Data availability statement

The raw data supporting the conclusions of this article will be made available by the authors, without undue reservation.

## Ethics statement

The studies involving human participants were reviewed and approved by Regional Ethics Committee South East Norway Document-id: 594040. Written informed consent to participate in this study was provided by the participants' legal guardian/next of kin.

## The Norwegian enhanced pediatric immunisation surveillance network

Håkon Bøås, Terese Bekkevold, Lise Beier Havdal, Anne-Marte Bakken Kran, Astrid Elisabeth Rojahn, Ketil Størdal, Sara Molvig Debes, Henrik Døllner, Svein Arne Nordbø, Bjørn Barstad, Elisebet Haarr, Liliana Vázquez Fernández, Britt Nakstad, Christopher Inchley, Truls Michael Leegaard, Elmira Flem.

## Author contributions

Statistical analyses: LH and HB. Drafting of manuscript: LH. All authors contributed to the article, approved the submitted version, conceptualization, study design, primary data acquisition, and revising manuscript for intellectual content.

## Funding

This work was supported by grants from the Research Council of Norway [240207/F20] and the South-Eastern Norway Regional Health Authority [2016007]. The study sponsors had no say in study design, collection, analysis or interpretation of data, the writing of the paper, or the decision to submit the paper for publication.

## Conflict of interest

EF was currently employed by Merck & Co., Inc., Kenilworth, New Jersey. The work for the current study was conducted by EF under the previous affiliation. The remaining authors declare that the research was conducted in the absence of any commercial or financial relationships that could be construed as a potential conflict of interest.

## Publisher's note

All claims expressed in this article are solely those of the authors and do not necessarily represent those of their affiliated organizations, or those of the publisher, the editors and the reviewers. Any product that may be evaluated in this article, or claim that may be made by its manufacturer, is not guaranteed or endorsed by the publisher.
