# Supplementary material for: Risk factors associated with severe disease in respiratory syncytial virus infected children under 5 years of age
Source: Front Pediatr. 2022 Aug 30;10:1004739. doi: 10.3389/fped.2022.1004739 (PMC9468371; doi:10.3389/fped.2022.1004739)
Supplement: Supplementary file 1 [file Table_1.DOCX]

**Supplementary data 1. Table.**

**ICD-10 codes and medical birth registry data used to identify comorbidities and risk factors in RSV infected children**

| **Disease** | **Norwegian Patient Registry ICD-10 Norwegian version** | **Medical Birth Registry of Norway** |
| --- | --- | --- |
| Prematurity | *Defined based on MBRN only* | Defined by gestational age |
| Trisomy 21 | Q90 Trisomy 21 | Trisomy 21 |
| Congenital heart disease | Q20 Congenital malformations of cardiac chambers and connections  Q21 Congenital malformations of cardiac septa  Q22 Congenital malformations of pulmonary and tricuspid valves  Q23 Congenital malformations of aortic and mitral valves  Q24 Other congenital malformations of heart  Q25 Congenital malformations of great arteries  Q26 Congenital malformations of great veins  I50 Congestive heart failure |  |
| Broncho-pulmonary dysplasia | P27 Bronchopulmonary dysplasia originating in the perinatal period |  |
| Chronic respiratory disease,  excluding asthma | E84 Cystic fibrosis  J43 Emphysema  J44 Other chronic obstructive pulmonary disease  J47 Bronchiectasis  Q32 Congenital malformations of trachea and bronchus  Q33 Congenital malformations of lung  Q34 Other congenital malformations of respiratory system  Q79.0 Congenital diaphragmatic hernia |  |
| Neuromuscular disease | G09 Sequelae of inflammatory disease of central nervous system  G10-14 Systemic atrophies primarily affecting the central nervous system  G35-G37 Demyelinating disease of the central nervous system  G40-G47 Episodic and paroxysmal disorders G70  G71-G73 Diseases of myoneural junction and muscle  G80-G83 Cerebral palsy and other paralytic syndromes  E71 Disorders of branched chain amino-acid metabolism and fatty-acid metabolism  E72 Other disorders of amino-acid metabolism  E75 Disorders of sphingolipid metabolism and other lipid storage disorders  E76 Disorders of glycosaminoglycan metabolism E77 Disorders of glycoprotein metabolism  E791 Lesch-Nyhan syndrome  E805 Crigler Najjar |  |
| Immunodeficiency | D46 Myelodysplastic syndromes  D57 Sickle-cell disorders  D61 Other aplastic anaemias  D70 Agranulocytosis  D71 Functional disorders of polymorphonuclear neutrophils  D73.0 Hyposplenism  D73.1 Hypersplenism  D73.2 Chronic congestive splenomegaly  D76 Certain diseases involving lymphoreticular tissue and reticulohistiocytic system  D80 Immunodeficiency with predominantly antibody defects  D81 Combined immunodeficiencies  D82 Immunodeficiency associated with other major defects  D83 Common variable immunodeficiency  D84 Other immunodeficiencies  B20-B24 Human immunodeficiency virus [HIV] disease   Z94 Transplanted organ and tissue status |  |
| Cancer | C00-C97 Malignant neoplasms |  |
