# Supplementary material for: Risk factors associated with severe disease in respiratory syncytial virus infected children under 5 years of age
Source: Front Pediatr. 2022 Aug 30;10:1004739. doi: 10.3389/fped.2022.1004739 (PMC9468371; doi:10.3389/fped.2022.1004739)
Supplement: Supplementary file 2 [file Table_2.DOCX]

**Supplementary data 2. Table:**

**Summary of variables in the analysis model for RSV severity, including the amount of data available for each variable, those with complete records, and crude odds ratio for being a complete case.**

|  | Available data; n=1096; N (%) | N (%) | Complete records; n=399; N (%) | Crude OR for being a complete case (95%CI) |
| --- | --- | --- | --- | --- |
| **Respiratory support** | 1075(98.1%) |  |  |  |
| yes |  | 202 (18.8%) | 103(25.8%) | 2.02(1.49-2.76) |
| no |  | 873 (81.2%) | 296(74.2%) |  |
| **Length of stay > 72 hours** | 978(89.2%) |  |  |  |
| yes |  | 317 (28.9%) | 145(36.3%) |  |
| no |  | 661 (60.3%) | 254(63.7%) | 1.35(1.03-1.77) |
| ***Demographic risk factors*** |  |  |  |  |
| **Age by age group** | 1096(100%) |  |  |  |
| <3months |  | 301 (27.5) | 159(39.9%) | ref |
| 3-5m |  | 181 (16.5) | 98(24.6%) | 1.05(0.73-1.53) |
| 6-11m |  | 170 (15.5) | 70(17.5%) | 0.63(0.43-0.91) |
| 1-2y |  | 297 (27.1) | 55(13.8%) | 0.20(0.14-0.29) |
| 2-5y |  | 147 (13.4) | 17(4.3%) | 0.12(0.07-0.20) |
| **Sex** | 1096(100%) |  |  |  |
| male |  | 615 (56.1) | 235(58.9%) |  |
| female |  | 481 (43.8%) | 164(41.1%) | 0.84(0.65- 1.07) |
| **Siblings** | 1031(94%) |  |  |  |
| yes |  | 766 (74.3%) | 301(75.4%) | 1.10(0.83-1.47) |
| no |  | 265 (25.7%) | 98(24.6%) |  |
| **Pets** | 1032(94%) |  |  |  |
| yes |  | 259 (25%) | 113(28.3%) | 1.33(0.99-1.73) |
| no |  | 773 (74.9%) | 286(71.7%) |  |
| **Daycare attendance** | 822(75%) |  |  |  |
| yes |  | 329 (40%) | 79(19.8%) | 0.17(0.12-0.23) |
| no |  | 493 (60%) | 320(80.2%) |  |
| ***Gestational risk factors*** |  |  |  |  |
| **Mothers age** | 1091(99.5%) |  | 428(100%) |  |
| Mean(SD) |  | 31.0(4.90) | 30.8(4.83) |  |
| For 1y increase |  |  |  | -0.01(-0.04-0.01) |
| **Smoking during pregnancy** | 974(88.8%) |  |  |  |
| yes |  | 50 (5.1%) | 14(3.5%) | 1.12(0.55-2.29) |
| no |  | 924 (94.9%) | 385(96.5%) |  |
| **Prematurity** | 1090(%) |  |  |  |
| GA >32 weeks |  | 1061 (97.3%) | 390(97.7%) |  |
| GA <32 weeks |  | 29 (2.7%) | 9(2.1%) | 0.77(0.35-1.71) |
| **Caesarean delivery** | 1091(99.5%) |  |  |  |
| yes |  | 222 (20.3%) | 78(19.6%) | 0.92(0.58-1.26) |
| no |  | 869 (79.7%) | 321(80.5%) |  |
| **Multiple gestation** | 1091(99.5%) |  |  |  |
| yes |  | 1035 (94.9%) | 384(96.2%) | 0.55(0.30-1.01) |
| no |  | 56 (5.1%) | 15(3.8%) |  |
| **Small for gestational age** | 1090(100%) |  |  |  |
| Z-Score>-1.28 |  | 1003 (92.0%) | 363(91.0%) |  |
| Z-score<-1.28 |  | 87 (8.0%) | 38(9.0%) | 1.24(0.80-1.94) |
| ***Comorbidity and dispositions*** |  |  |  |  |
| **Breastfeeding** | 739(67.4%) |  |  |  |
| yes |  | 693 (93.8%) | 370(92.7%) |  |
| no |  | 46 (6.2%) | 29(7.3%) | 1.49(0.80-2.76) |
| **Family history of atopy** | 738(67.3%) |  |  |  |
| yes |  | 125 (16.9%) | 51(12.8%) | 0.52(0.36-0.78) |
| no |  | 613 (83.1%) | 348(87.2%) |  |
| **Comorbidity** | 1096(100%) |  |  |  |
| yes |  | 94 (9%) | 29 (7.3%) | 0.76(0.48-1.20) |
| no |  | 1002 (91%) | 370 (92.3%) |  |
| **Respiratory support during neonatal period** | 1096(100%) |  |  |  |
| yes |  | 84 (7.7%) | 28 (7%) | 0.86(0.54-1.38) |
| no |  | 1012 (92.3%) | 371(93%) |  |
| **Viral Co-detection** | 1095 (99.9%) |  |  |  |
| yes |  | 76 (6.9%) | 24 (6%) | 0.79 (0.48-1.31) |
| no |  | 1019 (93.1) | 375 (94.0%) |  |
